# Phencyclidine and Scopolamine for Modeling Amnesia in Rodents: Direct Comparison with the Use of Barnes Maze Test and Contextual Fear Conditioning Test in Mice

**DOI:** 10.1007/s12640-018-9901-7

**Published:** 2018-04-21

**Authors:** Natalia Malikowska-Racia, Adrian Podkowa, Kinga Sałat

**Affiliations:** Department of Pharmacodynamics, Chair of Pharmacodynamics, Jagiellonian University Medical College, 9 Medyczna St., 30 - 688, Krakow, Poland

**Keywords:** Scopolamine, Phencyclidine, Barnes maze, Contextual fear conditioning paradigm, Freezing and grooming behavior, Mice

## Abstract

Nowadays cognitive impairments are a growing unresolved medical issue which may accompany many diseases and therapies, furthermore, numerous researchers investigate various neurobiological aspects of human memory to find possible ways to improve it. Until any other method is discovered, in vivo studies remain the only available tool for memory evaluation. At first, researchers need to choose a model of amnesia which may strongly influence observed results. Thereby a deeper insight into a model itself may increase the quality and reliability of results. The most common method to impair memory in rodents is the pretreatment with drugs that disrupt learning and memory. Taking this into consideration, we compared the activity of agents commonly used for this purpose. We investigated effects of phencyclidine (PCP), a non-competitive NMDA receptor antagonist, and scopolamine (SCOP), an antagonist of muscarinic receptors, on short-term spatial memory and classical fear conditioning in mice. PCP (3 mg/kg) and SCOP (1 mg/kg) were administrated intraperitoneally 30 min before behavioral paradigms. To assess the influence of PCP and SCOP on short-term spatial memory, the Barnes maze test in C57BL/J6 mice was used. Effects on classical conditioning were evaluated using contextual fear conditioning test. Additionally, spontaneous locomotor activity of mice was measured. These two tests were performed in CD-1 mice. Our study reports that both tested agents disturbed short-term spatial memory in the Barnes maze test, however, SCOP revealed a higher activity. Surprisingly, learning in contextual fear conditioning test was impaired only by SCOP.

**Graphical Abstract:**
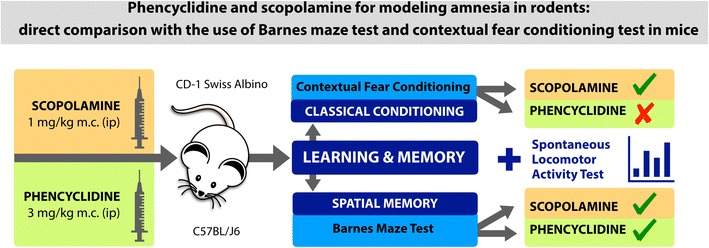
ᅟ

ᅟ

## Introduction

Memory deficits belong to the most frequent impairments that accompany psychiatric, neurological, or metabolic disorders, including Alzheimer’s disease (Tromp et al. 2015), epilepsy (Dupont 2015), diabetes mellitus (Sadanand et al. 2016), and others. Moreover, cognitive decline can be a manifestation of adverse effect of various drugs, for instance analgesics (Finnerup et al. 2015), or antiepileptic drugs (Javed et al. 2015). Because impaired memory significantly worsens patients’ quality of life, much effort has been made to discover and develop drugs with anti-amnesic properties.

In vitro assays have been found very useful for preliminary studies on learning and memory phenomena, but to fully investigate neurobiological aspects of learning processes and memory formation in living organisms, rodent models are involved as the critical path at the preclinical stage of research.

**Fig. 1:**
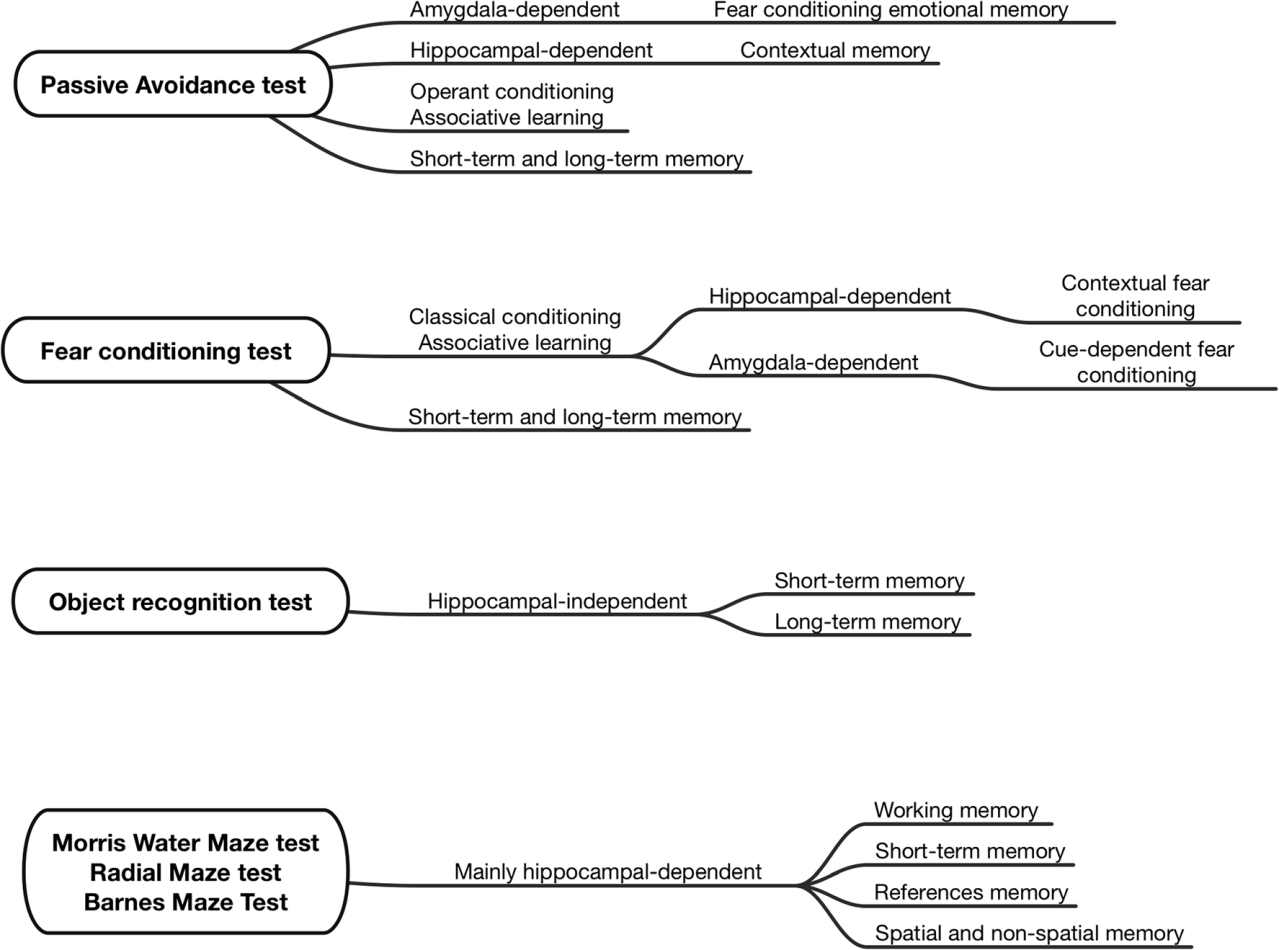
Most popular in vivo assays used to study various types of memory

In this area of experiments, several animal models mimicking cognitive decline, as well as numerous behavioral tests for its assessment have been introduced. These valuable research tools enable to study various types of memory (Fig. 1) (Blaser and Heyser 2015; Cho et al. 1999; Frankland et al. 1998; Malikowska et al. 2017; Podkowa et al. 2016; Sałat et al. 2016; Stiedl et al. 2000; Sunyer et al. 2007).

In our laboratory, we use passive avoidance (PA) task, Morris water maze (MWM), radial-arm water maze (RAWM), Barnes maze (BM), and novel object recognition (NOR) task to study various aspects of learning and memory processes and to discover compounds with potential anti-amnesic properties (Malikowska et al. 2017; Podkowa et al. 2016; Salat et al. 2015, 2017a; Wilcock et al. 2004). Although each of these tasks is burdened with some disadvantages, using them together can provide complementary results.

In our previous studies we utilized PA, MWM, and RAWM tests for the assessment of anti-amnesic properties of various biologically active compounds (Košak et al. 2016; Malikowska et al. 2017; Podkowa et al. 2016; Salat et al. 2015, 2017b). To induce memory deficits in mice, we used intraperitoneal injections of scopolamine (SCOP) or phencyclidine (PCP). These experiments confirmed pro-amnesic properties of both SCOP and PCP. However, it should be noted that the assessment of memory deficits was performed using tasks involving aversive stimuli, i.e., high-stress level conditions (PA: electric shock, and MWM, RAWM: water immersion and repeated swimming sessions) which are regarded as independent key factors responsible for the development of stress and depression (Kato et al. 2016). In rodents, water immersion with a subsequent measurement of depression-like behavior (‘immobility’) is known as the forced swim test (Porsolt et al. 1977). This assay is used to reveal antidepressant-like activity of drugs. At the same time, it points out that the assessment of animals’ cognition using only water mazes may be confounded. This issue is particularly important in the case of anticholinergic compounds, such as SCOP which are known for their antidepressant-like properties both in animals (Petryshen et al. 2016; Podkowa et al. 2016) and humans (Rigal et al. 2016). Also, testing conditions might significantly interfere with animals’ behavior, for example, an electric shock utilized to generate fear-motivated contextual learning and memory may additionally induce anxiety (Bentefour et al. 2016; Stiedl et al. 2000).

Taking these issues into consideration, in the present research we propose the use of BM to confirm potential usefulness of SCOP and PCP as tools to induce amnesia in rodents. This study complements our previous research on SCOP and PCP (Malikowska et al. 2017) and provides a deeper insight into chemically induced models and their effects on short-term spatial memory, contextual memory, as well as classical and instrumental conditioning. Tested doses of SCOP and PCP were selected based on our previous experiment (Malikowska et al. 2017; Salat et al. 2015) and available literature data (Bonito-Oliva et al. 2016; Harrison et al. 2010; Jiang et al. 2016; Klinkenberg and Blokland 2010; Komater et al. 2005; Oyamada et al. 2015).

The key advantage of BM over MWM or RAWM is that BM is a dry-land maze able to assess spatial learning without exposing animals to stress due to water immersion (Sunyer et al. 2007). The impact of SCOP on memory impairments is an area of extended studies. Although this muscarinic receptor antagonist is used to induce memory impairments (Klinkenberg and Blokland 2010; Haider et al. 2016; Jiang et al. 2016; Podkowa et al. 2016; Salat et al. 2015), there is also some bias about this agent, as the blockade of presynaptic muscarinic M2 receptors might increase the release of acetylcholine (Felder 1995; Mohr et al. 2015; Quirion et al. 1994). Hence, some authors propose the use of PCP as an alternative tool to induce learning and memory deficits in rodents.

As a part of the present study, we also assess pro-amnesic properties of intraperitoneally administered SCOP and PCP in the contextual fear conditioning paradigm (CFC). Fear conditioning is strongly associated with some psychiatric disorders like phobias, or post-traumatic stress disorder (PTSD). Recently, a strong link between PTSD and memory impairments has been shown by Zhu and colleagues (Zhu et al. 2017). These authors investigated memory impairments in PTSD-exposed rats, and they observed significant changes in prefrontal cortex, hippocampus, and amygdala of stressed animals. It has been also demonstrated that in patients suffering from PTSD hippocampal tissue—a key brain structure for memory formation, is significantly reduced (Bennett et al. 2016). Based on these studies, it was concluded that in PTSD fear generalization and abnormal fear responses conditioned by trauma are evoked and associative learning in PTSD patients could be extended and some cues apparently unrelated to traumatic events may evoke a strong fear response.

Of note, previous studies have shown that the delay or lapse of a reaction to conditioned stimuli (i.e., context), which is the source of fear, is not simple forgetting but an active process called fear extinction (Furini et al. 2014; Kaplan and Moore 2011). Thus, the use of CFC enables to achieve a more detailed insight into the effects of SCOP and PCP on classical conditioning.

## Materials and Methods

### Chemicals

SCOP hydrobromide (dose 1 mg/kg) and PCP hydrochloride (dose 3 mg/kg) were purchased from Sigma Aldrich (Poland). For the in vivo tests, they were prepared in 0.9% saline (Polfa Kutno, Poland) and were administered intraperitoneally (i.p.) 30 min before behavioral tests. Control mice received 0.9% saline (i.p.). In the BM paradigm all drugs tested were administered intraperitoneally 30 min before every acquisition (training) session which was held on days 1–4. On day 1, the administration of compounds tested was done after an additional adaptation period, but 30 min before training. Drugs were not injected on day 5 when the final examination was performed. In the CFC paradigm drugs were administered 30 min before conditioning on the first day of this test. No drugs were administered on day two. In the locomotor activity test, the administration of drugs was immediately followed by a 30-min-lasting adaptation period. Next, the final examination was conducted.

### Animals and Housing Conditions

Behavioral experiments were carried out at the Department of Pharmacodynamics, Faculty of Pharmacy, Jagiellonian University Medical College in Krakow. Eight-week-old male C57BL/6 J mice weighing between 18 and 22 g were purchased from a licensed animal breeding farm (Staniszewska, Ilkowice, Poland). Since there is evidence that C57BL/6 J mice show advantages over CD-1 mice in spatial learning and memory tasks which is due to their better visual skills as compared to Swiss Albino CD-1 mice (Patil et al. 2009), these mice were used in the BM task.

Male CD-1 Albino Swiss mice of the same age and body weight were purchased from the animal breeding farm at the Jagiellonian University, Faculty of Pharmacy in Krakow. These mice were used in the locomotor activity and CFC tests. The animals were housed in groups of six mice per cage at room temperature of 22 ± 2 °C, under light/dark (12:12) cycle. The animals had free access to food and water before experiments. The ambient temperature of the room and humidity (55 ± 5 °C) were kept consistent throughout the tests. For behavioral experiments the animals were selected randomly. Experimental groups consisted of 6–10 animals. The experiments were performed between 8 AM and 2 PM. Immediately after in vivo assays, the animals were euthanized by cervical dislocation. All procedures were approved by the Local Ethics Committee of the Jagiellonian University in Krakow, and the treatment of animals was in full accordance with ethical standards laid down in respective Polish and EU regulations (Directive No. 86/609/EEC).

### Behavioral Testing Paradigm

#### Barnes Maze Test

Spatial learning and memory in mice was assessed using BM (Harrison et al. 2010; Komater et al. 2005; Sunyer et al. 2007). This task was performed with the use of BM apparatus (Panlab-Harvard Apparatus, Spain). It consists of a circular, dry, open platform surface equipped with 18 holes around the perimeter of the platform and a small dark recessed chamber located under one of them. Visual extra-maze cues (pictures of colored geometric figures with contrasting background, placed at a distance 1 m from the platform) were provided to facilitate learning. Weak aversive stimuli (light (600 lx) and fan) were applied to increase animals’ motivation to escape from the circular platform (Paylor et al. 2001).

During the first 4 days of the experiment, the spatial acquisition trial was performed. On the first day of the test, an additional adaptation period preceded a proper trial. In this phase, mice were individually placed in a cylindrical black start chamber located in the middle of circular maze. Ten seconds later the mouse was released from the start chamber, the light and fan were turned on and the animal was gently guided to the escape box (a small dark recessed chamber located under the BM platform), where it remained for the next 2 min. This phase was not preceded by drugs injection.

In the spatial acquisition phase, each mouse was subjected to four trials daily with keeping 15-min intervals between each trial. Mice were injected with tested drugs or saline 30 min before the first trial on each day of the acquisition phase. In every trial, the mouse was placed in a cylindrical black start chamber in the middle of BM. After 10 s, the mouse was released from the start chamber, light and fan were turned on and the mouse was allowed to explore the maze for 180 s. During this phase, the number of primary errors (i.e., the number of errors before the mouse reached the escape box), total errors (i.e., the number of all errors made during 180 s of test), primary and total latencies (times) to find the escape box (i.e., time required to find and time required to enter the escape box, respectively) were measured. If the mouse did not find the escape box during 180 s, it was gently guided towards it. Immediately after the mouse entered the box, the light and fan were turned off and the mouse was allowed to stay in the box for 1 min. On day 5, the assessment of reference short-term memory was conducted (a drug-off probe trial). The escape box was removed and replaced by a closed area (plastic plate). Each mouse was placed in a cylindrical black start chamber. After its removal, the mouse was allowed to explore the maze for the next 90 s. Primary errors, total errors, and latency time to reach the former target (i.e., the escape box) location were recorded and analyzed (Sunyer et al. 2007).

#### Activity Monitoring

Activity monitoring was performed using activity cages (40 cm × 40 cm × 31 cm) supplied with I.R. horizontal and vertical beam emitters (Activity Cage 7441, Ugo Basile, Italy) connected to a counter measuring the number of light-beam crossings. The mice were i.p. injected with SCOP hydrobromide (1 mg/kg), PCP hydrochloride (3 mg/kg). Control mice were given saline. Then, the animals were placed in the activity cages located in a sound attenuated room for 30 min habituation period. Next, the final examination was performed. Software analysis enabled the measurement of ambulations and rearing during the next 30 min at 6-min intervals (Cartmell et al. 2000). Drugs or saline were administered 30 min before the final examination.

#### Contextual Fear Conditioning Paradigm

This test was performed following the methods described by three research groups: Stiedl et al. (2000), Brown et al. (2011) and Bentefour et al. (2016). Contextual fear conditioning was performed using apparatus (Panlab-Harvard Apparatus, Spain) which consists of a large white-painted illuminated compartment (26 cm × 26 cm × 34 cm) and a small black-painted compartment (13 cm × 7.5 cm × 7.5 cm) which are separated from each other by a guillotine gate (5 cm × 5 cm). The floor of the dark compartment enabled delivery of electric shocks.

On the first day of the experiment, 30 min after the injection of SCOP, PCP, or saline, every mouse was placed individually in a white compartment and was allowed to explore the whole apparatus for 2 min (the guillotine gate remained open). Afterwards, the mouse was gently moved into the dark compartment and guillotine gate closed. Then, three electric shocks (current intensity: 0.7 mA; stimulus duration: 3 s) at an interval of 1 min were delivered to the animals, except for the CFC electric shocks not-subjected control group, i.e., the mice of CFC electrics shocks not-subjected control group were allowed to explore the apparatus for 2 min, then they were gently moved to dark compartment, where they remained for the next 3 min and no electric shock was delivered. In contrast, electric shocks-subjected control was exposed to both exploration and an electric shock. Next, the mice were placed in their home cages. On the second day, neither SCOP nor PCP was administered. Mice were individually placed in the white compartment and were allowed to explore both compartments for 3 min (the guillotine gate was open). For this period, the duration of freezing behavior (i.e., complete immobility, excluding breathing and heart-beating movements), as an indicator of anxiety, was measured. Moreover, the duration of grooming behavior was also recorded.

#### Statistical Analysis

Data analysis of the results was carried out using GraphPad Prism software (v. 5, CA, USA). Numerical results from behavioral tests are expressed as mean ± SEM. For the statistical analysis, one-way analysis of variance (ANOVA) was used, followed by Dunnett’s post hoc comparison, or two-way repeated measures ANOVA, followed by Bonferroni’s multiple comparison. *P* < 0.05 was considered significant.

## Results

### Barnes Maze Test

**Fig. 2:**
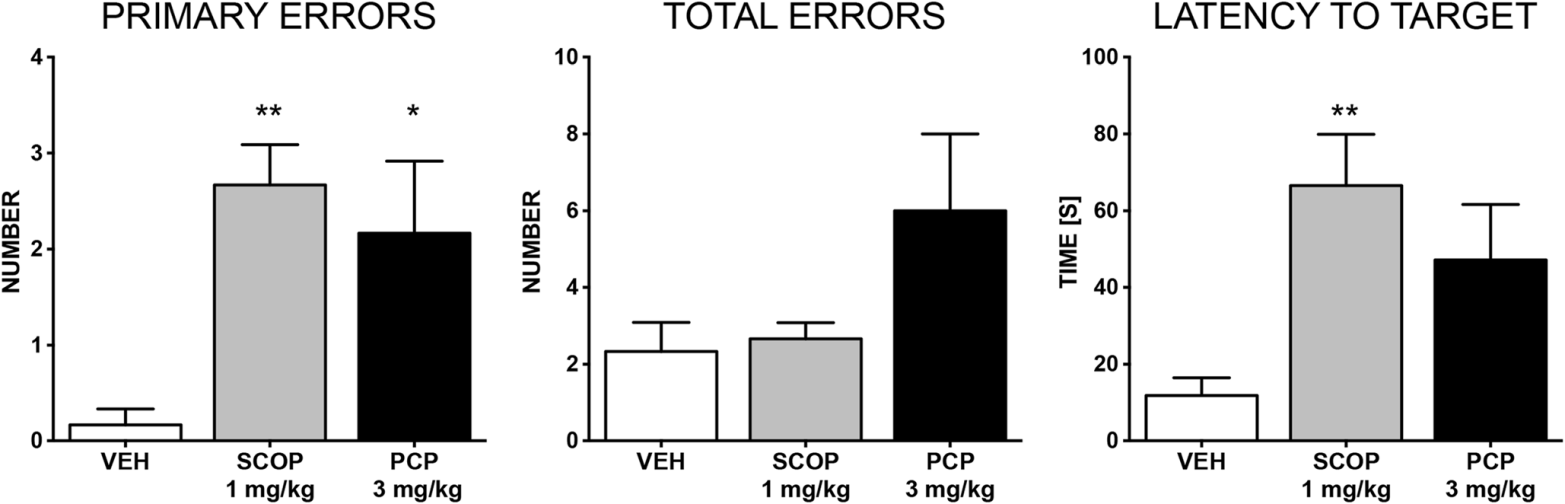
Effects of SCOP (1 mg/kg, i.p.) and PCP (3 mg/kg, i.p.) on the number of primary errors, total errors and latency to reach the former target (i.e. the escape box) location measured in BM test performed on day 5. Results are shown as the mean number of errors ± SEM and latency time [s] ± SEM to reach the former target (the escape box) location. Statistical analysis: one-way analysis of variance (ANOVA), followed by Dunnett’s multiple comparison. Significance vs. vehicle-treated group: *p < 0.05; **p < 0.01

**Fig. 3:**
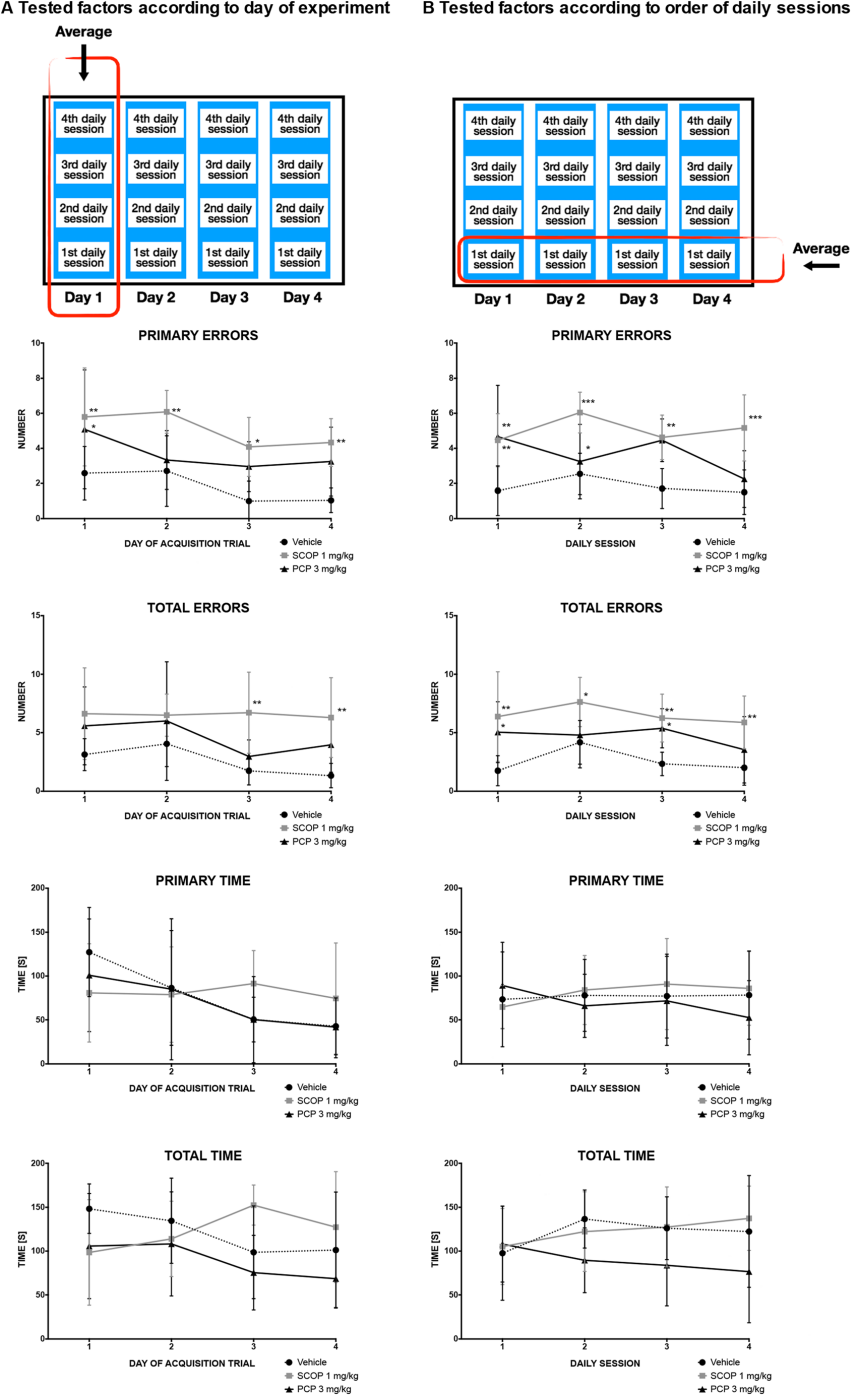
Effects of SCOP (1 mg/kg, i.p.) and PCP (3 mg/kg, i.p.) on working and short-term memory during the acquisition phase of the BM test, performed on days 1–4. Number of errors and latency time during consecutive days of experiment (i.e., averages of all trials performed on each day 1–4) (**a**), or consecutive daily sessions (i.e., the daily trials averaged across the four acquisition days; e.g., an average of trial 1 on acquisition days 1–4, an average of trial 2 on days 1–4, etc. (**b**). Results are shown as the mean number of errors ± SEM and latency time [s] ± SEM to enter or recognize the escape box. Statistical analysis: two-way repeated measures ANOVA, followed by Bonferroni’s multiple comparison. Significance vs. vehicle-treated group: *p < 0.05; **p < 0.01; ***p < 0.001

In this task, the influence of SCOP and PCP on short-term memory was assessed. Results of the probe trial and the acquisition trial are shown in Figs. 2 and 3, respectively. During the acquisition trial, primary errors were influenced both by factors, i.e., drug (*F* [2.15] = 16.41; *p* = 0.0002) and time (*F* [3.45] = 4.08; *p* = 0.012), however, overall interaction was considered not significant (*F* [6.45] = 0.45; *p* = 0.8436) (Fig. 3a). The administration of SCOP entailed a statistically significant increase in primary errors during each day of the acquisition phase (day 1: *p* < 0.01, day 2: *p* < 0.01, day 3: *p* < 0.05, day 4: *p* < 0.01) (Fig. 3a). In contrast to this, PCP significantly (*p* < 0.05) increased the number of primary errors only on day 1. Total errors were influenced only by drug administration (*F* [2.15] = 10.23; *p* = 0.0016). The injection of SCOP increased the number of total errors on days 3 (*p* < 0.01) and 4 (*p* < 0.01) of the acquisition trial (Fig. 3a). The comparison of daily scores of the vehicle-treated group and drug-treated groups showed no influence of SCOP or PCP on either primary, or total latency when average values in consecutive sessions were compared (i.e., daily trials 1–4 averaged across the four acquisition days). Primary errors were influenced by the drug factor (*F* [2.15] = 16.41; *p* = 0.0002), but not time factor (*F* [3.45] = 1.27; *p* = 0.2949) (Fig. 3b). Overall interaction was considered not significant (*F* [6.45] = 1.98; *p* = 0.0886). SCOP led to a statistically significant reduction of primary errors in the first (*p* < 0.01), second (*p* < 0.001), third (*p* < 0.01), and fourth (*p* < 0.001) sessions, whereas PCP caused a significant effect only in the first (*p* < 0.01) and third (*p* < 0.05) sessions. Also total errors were significantly influenced (drug effect: *F* [2.15] = 9.91; *p* = 0.0018; time effect: *F* [3.45] = 2.47; *p* = 0.0743), but overall interaction was not significant (*F* [6.45] = 0.7; *p* = 0.6476) (Fig. 3b). SCOP caused a statistically significant reduction of total errors in the first (*p* < 0.01), second (*p* < 0.05), third (*p* < 0.01) and fourth (*p* < 0.01) sessions, whereas PCP caused a significant effect only in the first (*p* < 0.05) and third (*p* < 0.05) sessions.

In the probe (drug-off) trial on day 5, primary errors were influenced by both SCOP and PCP (*F* [2.15] = 6.848; *p* = 0.0077). However, latency time to reach the former target (i.e., the escape box) location was significantly altered only in the SCOP-treated group (*F* [2.15] = 5.610; *p* = 0.0152).

### Activity Monitoring

**Fig. 4:**
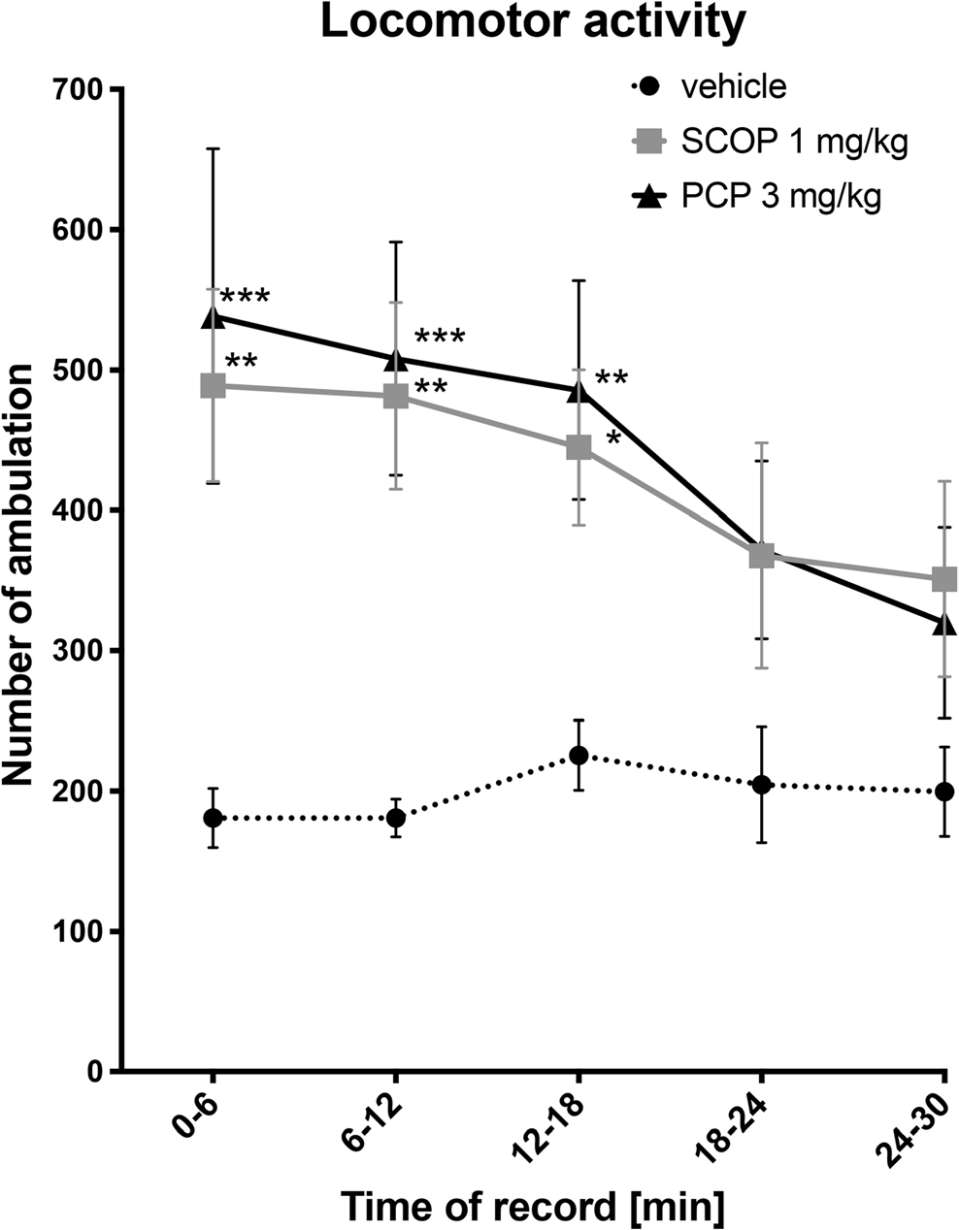
Influence of SCOP (1 mg/kg, i.p.) and PCP (3 mg/kg, i.p.) on animals’ locomotor activity. Results are shown as the mean number of ambulation (± SEM). Statistical analysis: two-way repeated measures ANOVA, followed by Bonferroni’s multiple comparisons. Significance vs. vehicle-treated group: *p < 0.05; **p < 0.01; ***p < 0.001

In this test, the number of ambulations was measured. Both time and drugs influenced the results in a statistically significant manner (*F* [4.76] = 6.86; *p* < 0.0001; *F* [2.19] = 6.53; *p* < 0.01, respectively). An overall interaction was considered significant (*F* [8.76] = 2.59; *p* < 0.05). Compared to the control group, SCOP and PCP caused statistically significant increases in animals’ locomotor activity only during the first 18 min of the test (Fig. 4).

### Contextual Fear Conditioning Paradigm

In this test, duration of freezing and grooming behavior was measured. Both, treatment and time affected freezing behavior in a statistically significant manner (drug effect: *F* [3.35] = 9.77; *p* < 0.0001; time effect: *F* [1.35] = 30.33; *p* < 0.0001) and the interaction was considered significant (*F* [3.35] = 9.98; *p* < 0.0001). On the first day of the test, almost none of the animals presented freezing behavior. A control group, that was not exposed to an electric stimulus, presented no freezing behavior on both days of the experiment. On the second day of the experiment, the control group subjected to electric shocks presented a marked and statistically significant prolongation of freezing behavior when compared to control mice not subjected to electric shocks in CFC procedure (significant at *p* < 0.0001). The SCOP-treated group presented a statistically significant reduction of freezing behavior when compared to control mice subjected to electric shocks in CFC (*p* < 0.0001). Interestingly, there was no effect of PCP on duration of freezing behavior, and these results correlated with those of electric shocks-subjected CFC control group (Table 1).

**Table 1:** Effect of intraperitoneal SCOP and PCP on the duration of freezing and grooming behavior measured in CFC paradigm. Results are presented as the mean duration of freezing or grooming behavior ± SEM for *n* = 9–10. Statistical analysis: two-way repeated measures ANOVA followed by Bonferroni multiple comparison test. Significance vs. control subjected to electric shocks in CFC; *p < 0.05, ****p < 0.0001

Groups	Day of testing	Control not subjected to electric shocks in CFC	Control subjected to electric shocks in CFC	SCOP (CFC mice)	PCP (CFC mice)
Freezing behavior	Day 1	0	0.2 ± 0.2	0	0.1 ± 0.1
Time [s] ± SEM Significance vs. electric shocks-subjected CFC control ****p < 0.0001					
	Day 2	0****	19.55 ± 5.276	0.5 ± 0.401****	24 ± 5.723
Grooming behavior	Day 1	0.750 ± 0.430	1.950 ± 0.545	2.350 ± 0.553	0.850 ± 0.548
Time [s] ± SEM Significance vs. electric shocks-subjected CFC control *p < 0.05					
	Day 2	3.250 ± 1.389	3.100 ± 1.329	7.150 ± 1.974*	1.000 ± 0.847

## Discussion

In the present study, we used a dry-land maze (i.e., the BM task) and CFC paradigm to assess memory-impairing potential of SCOP and PCP in mice. The doses of both agents (1 and 3 mg/kg, respectively) used in this research were selected based on our previous studies (Malikowska et al. 2017; Salat et al. 2015) and available literature data (Harrison et al. 2010; Jiang et al. 2016; Klinkenberg and Blokland 2010; Komater et al. 2005).

In our earlier research (Malikowska et al. 2017), the ability of SCOP, PCP, and biperiden (BIP) to induce amnesia in mice was compared. In these experiments, SCOP and PCP demonstrated a more prominent than BIP pro-amnesic activity; hence, these two drugs were chosen to assess their effect on short-term spatial memory in BM and CFC. In BM task, C57BL/6 J mice were used, because of their good visual skills required for spatial memory examination (Puzzo et al. 2014), while CD-1 mice were utilized in CFC paradigm.

Many researchers indicate that there are two parameters crucial for BM, namely: primary latency (time) and primary errors, that is time required and number of errors made before the animal reaches the target—i.e. the escape box (Sunyer et al. 2007). The administration of SCOP resulted in the increase in both parameters, i.e., primary errors and primary latency (time). In other behavioral tests, i.e., MWM and RAWM corresponding results for SCOP used at the same dose were obtained (Podkowa et al. 2016; Salat et al. 2015). Furthermore, both SCOP and PCP were also highly active in a fear-conditioned behavioral test assessing contextual memory, namely, the passive avoidance task (Malikowska et al. 2017).

Voluntary entrance into the electric shock-associated area, which is directly measured by PA test is identified with an instrumental conditioning, whereas involuntary location could be more associated with a classical conditioning—there is an aversive enhancement of context, but not mouse reaction (i.e., entrance to the shock-associated area). It is confirmed by our previous study where the observed effects were different from these currently obtained, i.e., in the PA test PCP strongly impaired both contextual memory and instrumental conditioning. In contrast to this, in the current experiment only SCOP interfered with place-conditioning, which confirmed that both these tests involve different types of learning. To achieve the most accurate comparison, we decided to perform contextual fear conditioning with the use of the PA apparatus, with a view to keep the constant conditions of both tests.

This previous study (Malikowska et al. 2017) revealed that PCP might be slightly more active than SCOP but both studied compounds interfered with associative learning related to instrumental conditioning which is a source of avoidance behavior observed in the PA task (Furini et al. 2014). Following other researchers (Furini et al. 2014; Kaplan and Moore 2011) and considering learning extinction as an active process that involves long-term potentiation and long-term depression, the stronger negative effect of PCP, a NMDA receptor antagonist in the PA task remains unclear.

To investigate further this issue, we have also assessed the influence of SCOP and PCP on fear conditioning using the CFC test. Interestingly, in this experiment, SCOP was more active than PCP. SCOP-treated mice did not associate the context with aversive stimuli, and presented the same degree of anxiety-related behavior (measured as duration of freezing behavior) as vehicle-treated group not exposed to electric stimulus. Thus, it could be assumed that SCOP may alleviate contextual fear. On the other hand, the SCOP-injected group was the only one that presented a strong prolongation of grooming time, which might be a manifestation of increased stress and anxiety (Kalueff and Tuohimaa 2005; Sałat et al. 2016). These data remain apparently inconsistent, i.e., SCOP in the same test reduced contextual fear measured as freezing behavior and enhanced self-grooming that is usually associated with stress. Intensified grooming may be a result of increased locomotor activity and stereotypies; however, both SCOP and PCP are known to induce these effects, so it does not explain the difference observed (Haller et al. 2005, Laviolette et al. 2000). More recent data indicate that the correlation between situational aversiveness and intensity of grooming is represented by inverted U-shaped function (Fernandez-Teruel and Estanislau 2016; Song et al. 2016). According to these reports, both low and high level of aversiveness may result in reduced self-grooming. Strongly intensified anxiety/stress is thought to induce freezing behavior and it precludes grooming at the same time. Otherwise, in moderately stressful situations, the grooming behavior reaches the highest intensity. Interestingly, it is usually associated with a novel or changing environment and this might be a potential explanation for the results obtained in the CFC test—increased grooming observed in SCOP-treated mice may be a consequence of moderate stress in SCOP-treated memory-impaired mice which consider the compartment of CFC apparatus as the novel one. It suggests that SCOP-treated mice, in contrary to PCP-treated mice, did not remember the first exposition to CFC apparatus, which is further confirmed by reduced contextual fear.

A negative effect of PCP on memory is widely known (Morris 2013; Vyklicky et al. 2014; Jones et al. 1990). In the present research, in the probe trial of BM, PCP influenced only primary errors, whereas it increased the number of total errors insignificantly. In BM task, PCP compared to SCOP was less effective. PCP did not increase either primary or total time to reach the escape box and this suggests that it might impair only the quality of memory, but not the speed of recall. Alternatively, this effect might be a result of psychomotor stimulation caused by PCP (Figs. 3 and 4). It was also reported that the blockade of NMDA receptors is involved in fear conditioning and reduces fear in rats (Gilmartin and Helmstetter 2010; Laurent and Westbrook 2009; Zimmerman and Maren 2010). However, it should be noted that in these studies a competitive antagonist of NMDA receptors (APV) was used, whereas we used PCP which is an uncompetitive channel blocker. Furthermore, additional properties of PCP, such as inhibition of nicotinic acetylcholine receptors (Thomsen et al. 2009; Hashimoto et al. 2006), AMPA receptors (Katayama et al. 2007), and interaction with the dopaminergic system (Seeman et al. 2009) might also interfere with learning and memory processes. It was also demonstrated that the enhancement in NMDA-mediated transmission increases fear response (Yamada et al. 2009). Furthermore, the biological effects of NMDA receptor antagonists result from their prevalence in the central nervous system (Vyklicky et al. 2014). In line with the results obtained in CFC paradigm, the administration of PCP less impaired classically conditioned reaction to electric stimulation. Thus, the above-mentioned additional features of PCP might act as a compensating mechanism. It might mean that SCOP is a stronger pro-amnesic agent and it impairs not only the quality of re-called information but also the speed of recall. Of note, the last injections of SCOP and PCP were made 24 h before the final test. This suggests that the observed increased locomotion rather did not directly affect the results obtained in BM task.

The comparison of the results from consecutive days of the acquisition trial confirmed a stronger activity of SCOP than PCP in BM. Starting from the first day, an increase in primary and total errors was observed. It indicates that the injection of SCOP impaired learning abilities and memory. However, starting with the third day of learning trials, a reduction in primary errors, but not total errors, occurred. Since on the third day, a similar reduction of primary and total errors was noticed in the control group, it could be assumed that after the second day of memory acquisition trial a specific strengthening of memory processing might occur. Of note, for PCP this tendency was noticed only if total errors were counted (Fig. 3a). This preliminary observation seems very interesting but more detailed studies are needed. If daily results from the acquisition trial are compared, none of the agents tested significantly influenced either primary or total time. This analysis was done in order to evaluate if mice after a 24-h break could immediately recall the way to the escape box and if a single or repeated reminding the way to target facilitates memory recall. Simultaneously, there was an insignificant gradual reduction of recorded parameters in vehicle-treated and PCP-treated groups, which might indicate that SCOP-treated mice were less able to construct spatial memory. Because primary time was more strongly affected, it can be assumed that the mice were not motivated enough to enter the escape box to avoid aversive stimuli. Interestingly, in the SCOP-treated group a prolongation of both time factors since the second day was observed, which might indicate the development of memory impairments and reduction in subjective fear of stimulus.

Significant alterations of locomotor activity of mice might interfere with the results obtained in BM task. For instance, increased locomotor activity might lead to the increased number of errors, which falsely increases this parameter. Both SCOP and PCP significantly increased locomotor activity of mice during the first 18 min of the testing period. Thus, if changes observed for primary and total errors were false positive, then for every first daily session of the acquisition trial, we should observe a strong increase in primary and total errors, and this effect should be immediately reduced in the second daily session. However, this was not observed in our study, so the results from all sessions were taken for the statistical analysis.

To conclude, the present study indicates that in the BM task SCOP is a stronger inductor of spatial memory impairments as compared to PCP. Moreover, SCOP appears to be a more potent learning-distractor when the process of classical conditioning is involved. Results of this study taken together with our previous results (Malikowska et al. 2017) indicate that SCOP impairs instrumental conditioning (i.e., avoidance learning), alleviates classical conditioning (i.e., contextual fear), whereas PCP interferes only with instrumental one (i.e., avoidance learning) (Malikowska et al. 2017).

## Abbreviations

SCOP: Copolamine
PCP: Phencyclidine
BM: Barnes maze test
CFC: Contextual fear conditioning
